# COVID-19 after two years: trajectories of different components of mental health in the Spanish population

**DOI:** 10.1017/S2045796023000136

**Published:** 2023-04-17

**Authors:** I. Bayes-Marin, M. Cabello-Toscano, G. Cattaneo, J. Solana-Sánchez, D. Fernández, C. Portellano-Ortiz, J. M. Tormos, A. Pascual-Leone, D. Bartrés-Faz

**Affiliations:** 1 Departament de Medicina, Facultat de Medicina i Ciències de la Salut – Campus Clínic, Universitat de Barcelona, Barcelona, Spain; 2 Institut de Recerca Biomèdica August Pi i Sunyer (IDIBAPS), Barcelona, Spain; 3 Institut Guttmann, Institut Universitari de Neurorehabilitació adscrit a la Universitat Autónoma de Barcelona, Barcelona, Spain; 4 Fundació Institut d'Investigació en Ciències de la Salut Germans Trias i Pujol, Badalona, Spain; 5 Instituto de Salud Carlos III, Centro de Investigación Biomédica en Red de Salud Mental, CIBERSAM, Monforte de Lemos 3-5, Pabellón 11, 28029, Madrid, Spain; 6 Serra-Húnter fellow. Department of Statistics and Operations Research (DEIO), Universitat Politècnica de Catalunya ⋅ BarcelonaTech (UPC), 08028 Barcelona, Spain; 7 Institute of Mathematics of UPC – BarcelonaTech (IMTech), 08028 Barcelona, Spain; 8 Hinda and Arthur Marcus Institute for Aging Research and Deanna and Sidney Wolk Center for Memory Health, Hebrew SeniorLife, Harvard Medical School, Boston, MA, USA; 9 Department of Neurology, Harvard Medical School, Boston, MA, USA

**Keywords:** COVID-19, growth mixture models, mental health, trajectories

## Abstract

**Aims:**

Our study aimed to (1) identify trajectories on different mental health components during a two-year follow-up of the COVID-19 pandemic and contextualise them according to pandemic periods; (2) investigate the associations between mental health trajectories and several exposures, and determine whether there were differences among the different mental health outcomes regarding these associations.

**Methods:**

We included 5535 healthy individuals, aged 40–65 years old, from the Barcelona Brain Health Initiative (BBHI). Growth mixture models (GMM) were fitted to classify individuals into different trajectories for three mental health-related outcomes (psychological distress, personal growth and loneliness). Moreover, we fitted a multinomial regression model for each outcome considering class membership as the independent variable to assess the association with the predictors.

**Results:**

For the outcomes studied we identified three latent trajectories, differentiating two major trends, a large proportion of participants was classified into ‘resilient’ trajectories, and a smaller proportion into ‘chronic-worsening’ trajectories. For the former, we observed a lower susceptibility to the changes, whereas, for the latter, we noticed greater heterogeneity and susceptibility to different periods of the pandemic. From the multinomial regression models, we found global and cognitive health, and coping strategies as common protective factors among the studied mental health components. Nevertheless, some differences were found regarding the risk factors. Living alone was only significant for those classified into ‘chronic’ trajectories of loneliness, but not for the other outcomes. Similarly, secondary or higher education was only a risk factor for the ‘worsening’ trajectory of personal growth. Finally, smoking and sleeping problems were risk factors which were associated with the ‘chronic’ trajectory of psychological distress.

**Conclusions:**

Our results support heterogeneity in reactions to the pandemic and the need to study different mental health-related components over a longer follow-up period, as each one evolves differently depending on the pandemic period. In addition, the understanding of modifiable protective and risk factors associated with these trajectories would allow the characterisation of these segments of the population to create targeted interventions.

## Introduction

The COVID-19 pandemic posed an extraordinary health, social and economic challenge to the world. Due to the rapid spread of the virus, governments had to implement restrictive policies such as lockdowns or stay-at-home orders (COVID-19 Mental Disorders Collaborators, 2021). Although these restrictive policies varied between countries, they affected people's daily lives globally, in terms of their work, livelihood, leisure activities and social interactions (Prati and Mancini, 2021). In the case of Spain, in the two years following the start of the pandemic, different containment measures were put into place, interleaving periods of strict lock-down confinement (e.g., home confinement, closure of schools and businesses, use of facemasks outdoors/indoors) with those of more relaxed measures (progressive return to work, the opening of restaurants and shops, use of facemasks only in some enclosed spaces, etc.) (Red Nacional de Vigilancia Epidemiológica. Instituto de Salud Carlos III, 2022).

A large body of knowledge has been generated regarding the impact of the pandemic and confinement in relation to mental health (Salari *et al*., 2020; Prati and Mancini, 2021; Wu *et al*., 2021). Whether through cross-sectional or longitudinal studies, it has been reported prevalence rates or mean scores of depressive or anxiety symptoms, assuming that the response to the pandemic is homogeneous, i.e., the same among individuals (Shevlin *et al*., 2023). In contrast, a systematic review based on longitudinal studies declared that the effect of lockdowns on depression and anxiety was small and significant, but also highly heterogenous (Prati and Mancini, 2021). Similarly, a meta-review of mental health during the COVID-19 pandemic, found an increase of mental health problems from 20 to 36%, but also a high heterogeneity among studies (de Sousa *et al*., 2021). It is worth mentioning that this evidence come from studies carried out at most up to one year after the pandemic, with a lack of studies that have analysed longer-term consequences on mental health. According to Taylor (2019), pandemics are dynamic events and as such their reactions were likely to vary over time (Taylor, 2019). For this reason, the results should be contextualised at different times of the pandemic and the events occurring in each period. In addition, in order to evaluate change from pre-pandemic status, baseline information is needed, and this condition has been less available in the performed research (Ahrens *et al*., 2021; Ellwardt and Präg, 2021; Pierce *et al*., 2021).

In agreement with the assumption that psychological adjustment in front of an adverse event is heterogeneous and may vary over time, different studies have been carried out on mental health trajectories (Ahrens *et al*., 2021; Batterham *et al*., 2021; Ellwardt and Präg, 2021; Joshi *et al*., 2021; Pellerin *et al*., 2021; Pierce *et al*., 2021; Saunders *et al*., 2021; Shilton *et al*., 2021). Most of these studies identified trajectories based on depression and anxiety symptoms measures, using individual-centred statistical techniques, as growth mixture models (GMM) or latent class growth analysis. These techniques rely on the assumption that individuals can be assigned to homogeneous subgroups (i.e., distinct trajectories) based on similarities on given outcomes (Nguena Nguefack *et al*., 2020). The abovementioned investigations identified from two (Joshi *et al*., 2021) to five trajectories of depression or/and anxiety symptoms (Ahrens *et al*., 2021; Batterham *et al*., 2021; Ellwardt and Präg, 2021; Pellerin *et al*., 2021; Pierce *et al*., 2021; Saunders *et al*., 2021; Shilton *et al*., 2021). In general terms, the results showed that a large proportion of the sample was classified in a stable trajectory over time (called ‘resilient trajectory’), while a smaller proportion showed worse scores or worsening over the follow-up period (‘chronic’ and ‘deteriorating trajectories’) (Ahrens *et al*., 2021; Batterham *et al*., 2021; Ellwardt and Präg, 2021; Joshi *et al*., 2021; Pellerin *et al*., 2021; Pierce *et al*., 2021; Saunders *et al*., 2021; Shilton *et al*., 2021). These results support the model put forward by Bonanno (2004), which argued that resilience is extremely common, finding higher proportions in the so-called ‘resilient’ trajectory, where hardly any changes were observed throughout the follow-up in the face of a stressor (Bonanno, 2004).

Nevertheless, these studies focused on psychological distress as outcome measure, using mostly sociodemographic variables, and in some cases personality (Saunders *et al*., 2021), loneliness (Ahrens *et al*., 2021; Shevlin *et al*., 2023), coping strategies (Joshi *et al*., 2021; Lin *et al*., 2021; Pellerin *et al*., 2021) and subjective well-being variables (Pellerin *et al*., 2021) as predictors of these trajectories. According to Keyes *et al*. (2020), mental health is a conjunction of emotional (positive and negative affect and psychological distress), psychological (positive functioning variables, as meaning in life, personal growth, autonomy and environmental mastery) and social wellbeing (social integration, social contribution and social acceptance), being more than just the absence of psychopathology (Keyes *et al*., 2020). Accordingly, it might be hypothesised that we could find changes in these other components of mental health. For example, Baños *et al*. (2022) found in a sample of Spanish residents that the scores on positive functioning variables (meaning in life, gratitude, resilience, compassion and life satisfaction) worsened from the beginning of the lockdown, whereas emotional distress improved by the end of the first Spanish state of alarm (June 21st, 2020) (Baños *et al*., 2022). Thus, an in-depth study of the impact of the COVID-19 pandemic on mental health should not be limited to its effect on psychological distress, but on the different components of wellbeing affecting mental health.

Likewise, people classified into different trajectories differed in terms of several predictors at baseline. As reported in previous research, being younger, female, reporting lower income, less education and having a previous mental health diagnosis, were factors consistently associated with ‘chronic’ and ‘worsening’ trajectories (Pierce *et al*., 2021; Saunders *et al*., 2021; Shilton *et al*., 2021). Fewer research studies examined modifiable determinants associated with these mental health patterns such as emotion regulation, coping strategies and locus of control (Ahrens *et al*., 2021; Joshi *et al*., 2021; Shilton *et al*., 2021).

Altogether, the study of the impact of the pandemic on mental health should take into account the heterogeneity of responses to a crisis situation. Prevalence or incidence rates would not be sufficient to estimate its impact. In this sense, the study of mental health trajectories over a long follow-up would make it possible to identify subgroups of the population in a situation of greater vulnerability, as well as to visualise the most critical moments of the pandemic. Furthermore, the understanding of modifiable protective and risk factors associated with these trajectories would allow the characterisation of these segments of the population to create targeted interventions. The resulting body of knowledge would have considerable practical implications for pressing public health efforts.

Therefore, this study aimed to (1) identify trajectories based on different mental health components (emotional, psychological and social wellbeing) during a two-year follow-up of the COVID-19 pandemic, and contextualise them according to relevant events in each pandemic period; (2) investigate the associations between mental health trajectories and sociodemographic, personality, coping, subjective well-being and lifestyles variables, and to determine whether there were differences among the different mental health outcomes regarding these associations.

## Method

### Study design and participants

Middle- aged volunteers (40 to 65 years) participating in the Barcelona Brain Health Initiative (BBHI), an ongoing prospective longitudinal study that aims to understand and characterise the determinants of brain health maintenance, were invited to participate in the current study. Briefly, BBHI study participants are community-dwelling individuals, free from any self-reported neurological or psychiatric diagnosis at the time of the recruitment, who answer annual questionnaires regarding demographic, socio-economic, self-perceived health and lifestyles (general health, physical activity, cognitive ability, socialisation, sleep, nutrition and vital plan) information. The BBHI recruitment took place in 2017 through an intensive dissemination campaign including conferences, radio and TV interviews and social media advertisements. For further details of the cohort and study protocol see Cattaneo *et al*. (Cattaneo *et al*., 2018).

**Figure 1.**
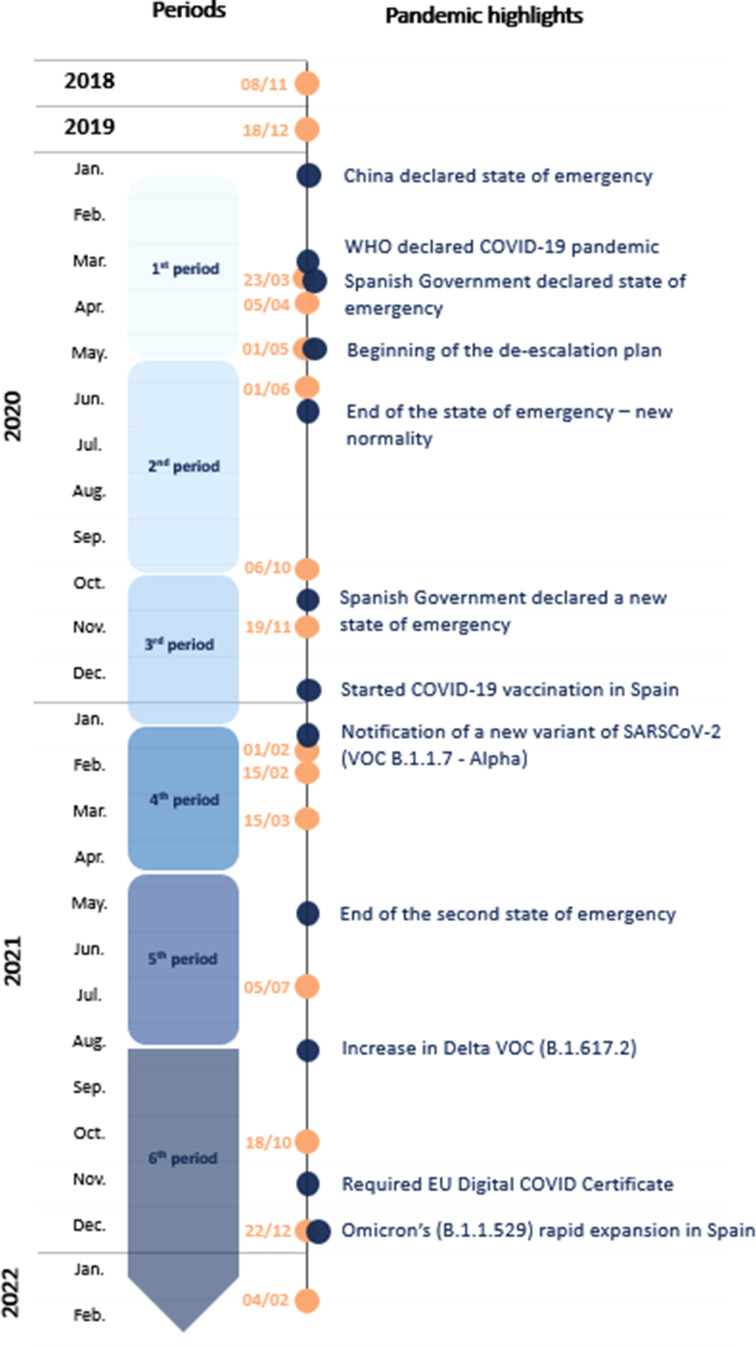
Timing of data acquisition and periods relative to the development of the COVID-19 pandemic in Spain. *Note:* Timeline showing the periods covered by the present study, according to the epidemic periods in Spain, as defined by the national epidemiological surveillance network of the Carlos III National Health Institute. Questionnaires launching is presented with orange dots, whereas blue dots represent relevant highlights of the pandemic.

The present work refers to a BBHI sub-study designed to investigate mental health during the COVID-19 pandemic (Bartrés-Faz *et al*., 2021; Pascual-Leone *et al*., 2021). BBHI participants who had completed the annual questionnaires before the COVID-19 widespread were invited to participate in subsequent brief evaluations (March, April, June and October 2020, March, July and October 2021 and February 2022) during the different periods of the COVID-19 pandemic (See Fig. 1). In this sub-study, several measures regarding mental health, subjective well-being, quality of life and coping strategies, were included to explore the effects of the pandemic on health and well-being.

In the present study, we included both the annual general follow-up questionnaires and the COVID-19 assessments, considering the observations two years before the pandemic (2018 and 2019 annual questionnaires) as baseline data. We decided not to include the 2017 annual questionnaire as we considered the information from two points before the pandemic as a good baseline on the individual's mental health status.

Figure 1 summarises the periods covered by our study (from early 2018 to February 2022), highlighting the time points when the questionnaires were launched (orange dots), the relevant highlights of the pandemic (blue dots) and their correspondence with the epidemic periods established by the national epidemiological surveillance network of the Carlos III National Health Institute (Red Nacional de Vigilancia Epidemiológica. Instituto de Salud Carlos III, 2022). These periods were defined by this national epidemiological surveillance network by analysing the evolution of incidence rates in the Spanish population.

The study was approved by the Catalan Union of Hospitals ethics committee [Unió Catalana d'Hospitals] (approval references: CEIC 17/06 and CEI 18/07). Moreover, written informed consent was obtained from all participants in accordance with the Code of Ethics of the World Medical Association (Declaration of Helsinki).

### Measures

#### Outcomes

According to Keyes *et al*. (2020) definition of mental health, we selected different variables as proxies for the emotional, psychological and social components. This selection was made according to the availability of longitudinal measures including baseline data and similarity to the constructs assessed (Keyes *et al*., 2020).

#### Emotional

To assess psychological distress, we used the Patient Health Questionnaire 4 items (PHQ-4) (Kroenke *et al*., 2009), a screening and accurate measurement of core symptoms or signs of depression (‘be bothered by little interest or pleasure in doing things’, ‘be bothered by feeling down, depressed, or hopeless’) and anxiety (‘feeling nervous, anxious or on edge’, ‘be bothered by not being able to stop or control worrying’). Participants were asked to indicate how often they have been bothered by four possible symptoms in the last 2 weeks, rated 0 ‘not at all’, 1 ‘several days’, 2 ‘more than half the days’, or 3 ‘nearly every day’. A score of six or higher represent the cut-off point for a potential case of depression/anxiety (Kroenke *et al*., 2009). However, in our analyses, we used the continuous form where higher scores mean greater psychological distress.

#### Psychological

This domain was constituted by ‘personal growth’, one of the positive functioning variables extracted from the Ryff Psychological wellbeing scale (SPWB) (Ryff, 1995; Ryff and Keyes, 1995). SPWB measure consists of 39 items, constituted by six sub-scales evaluating six aspects of positive functioning. Participants are asked to indicate how accurately each item describes themselves by rating on a 5-level Likert scale ranging from 1 ‘least like me’ to 5 ‘most like me’. Higher scores indicate better positive functioning. In particular, ‘personal growth’, is constituted by seven items and refers to one's openness to new experiences and growth.

#### Social

Keyes' social wellbeing definition includes different factors of the subjective evaluation of personal life circumstances and functioning in society, such as social contribution, integration, actualisation, acceptance and coherence. In the present study, we used the UCLA 3-Item Loneliness Scale (Rico-Uribe *et al*., 2016), as a proxy measure of social well-being. The UCLA items are related to social integration since refer to the feeling of being excluded or isolated from others. (Rico-Uribe *et al*., 2016).In this brief questionnaire, respondents were asked how often they felt that they: lacked companionship, were left out, and were isolated from others, on a 3-level Likert scale coded from 1 ‘hardly ever’, to 3 ‘often’. Higher scores indicate greater loneliness.

### Exposures

We included other variables, such as sociodemographic, self-perceived quality of life and health, lifestyles related to health, among other psychological measures to characterise the mental health trajectories.

The following sociodemographic variables were considered: sex (male/female), age (continuous), current marital status (single, married, divorced, widowed), living alone (yes/no), educational level (primary or less, secondary, higher education), occupation (employed, unemployed, retired), monthly family income (<1000€, 1000–2000€, 2000–5000€, >5000€), and if the person lives in a town or in a city (town/city).

Furthermore, to evaluate self-perceived general health and cognitive function we used the Patient-Reported Outcomes Measurement Information System (PROMIS) of global health (Ader, 2007) and the PROMIS Applied Cognition – General Concerns scale (Fieo *et al*., 2016), respectively. The PROMIS Global Health is composed by ten items representing five domains (physical function, pain, fatigue, emotional distress, social health) that are used to assess global physical health. Concerning the cognitive function scale, is comprised by eight items assessing self-reported cognitive troubles or deficits. In both measures, higher scores mean better general health and better cognitive functioning.

In addition, we included some variables related to lifestyles, as sleeping problems and tobacco consumption. Sleeping problems (i.e., difficulty to fall asleep, wake up at night) were assessed through the Jenkins Sleep Evaluation Questionnaire, a 4-item questionnaire with scores ranging from 0 (no sleep problems) to 20 (most sleep problems) (Jenkins *et al*., 1988). Moreover, tobacco coded as *yes/no* was included in our analyses.

We also considered the big five personality traits (extraversion, emotional stability, agreeableness, conscientiousness and openness to experience), assessed via the International Personality Item Pool (Goldberg, 1992). Resilience and coping strategies were evaluated with the Brief Resilience and Coping Scale (BRCS) (Sinclair and Wallston, 2004), where higher scores mean better resilience and coping ability.

Related to this, we added the Engaged Living Scale to assess an engaged response style (Trompetter *et al*., 2013), and three of the six scales from the SPWB: autonomy (a sense of autonomy in thought and action), environmental mastery (the ability to manage complex environments to suit personal needs and values) and positive relations with others (the establishment of quality ties to other) (Ryff, 1995). For each of these scales, higher scores are indicative of better functioning.

Furthermore, perceived stress (the Perceived Stress Scale (Cohen *et al*., 1983)) was included as a continuous measure. In this case, higher scores mean worse level of that construct.

### Statistical analysis

We performed a descriptive analysis of the exposures at baseline. Continuous variables were described by mean ± s.d. values, while categorical variables were presented by the absolute number of individuals and its corresponding percentage (%) within the sample. We considered the information extracted from the annual questionnaires before the COVID-19 pandemic as the baseline. In the case of the variables ‘resilience and coping strategies’ and ‘perceived stress’, no pre-pandemic data were available. These two variables were collected in different assessments and to increase the sample size, we considered as baseline the first available observation of each subject on each of these two variables.

To identify mental health trajectories, we first fitted multiple general mixed effects models for each outcome (psychological distress, personal growth and feelings of loneliness), to explore the extent of between-individual heterogeneities (as also recommended in (Herle *et al*., 2020)). These models separately can allow the estimation of random intercepts, random slopes or both. In this line, these models were compared using a Chi-squared test to find the best design option and do model selection (i.e., the one with the lowest residual sum of squares) (online Supplementary material, Table 2). Second and guided by the results in the previous step, we fitted a GMM with random intercepts and slopes for each outcome to classify individuals into latent trajectories based on their score on the outcome variables without covariates (Nagin and Tremblay, 2005; Berlin *et al*., 2014; Nagin, 2014). The number of trajectories was determined by analysing group models from 1 to 5 trajectories. According to the Bayesian information criterion (BIC) and the Akaike information criterion (AIC), where the lowest value indicates the better fit, the optimal model was selected (Schwarz, 1978; Akaike, 1998). Moreover, average posterior probabilities above 0.70 were considered as indicators of optimal fit (Tein *et al*., 2013; Nylund-Gibson and Choi, 2018). Trajectories sample size was also considered since inadequate sample size (lower than 5% can lead to convergence problems, insufficient power to identify classes and changing solutions) (Nylund-Gibson and Choi, 2018). The time variable within the GMM was ‘months of the study’, although for a clearer presentation of the results, we used the pandemic periods established by the national epidemiological surveillance network of the Carlos III National Health Institute when plotting these.

Then, multiple imputation by chained equations was used to deal with missing data in some of the exposures (online Supplementary Table 3), assuming missing-at-random (MAR), which can handle variables of varying types (Lepkowski *et al*., 2001; van Buuren, 2007). The imputation model included the outcome (i.e., trajectories membership) and all the variables described in the exposures section, generating 20 imputed datasets (He, 2010). To check imputation quality, we compared imputed and observed data using density and stripplots of van Buuren and Greenacre (van Buren and Greenacre, 2018) (online Supplementary Figs 1 and 2, respectively).

To study the relationship between latent trajectory membership and the described exposures, we first fitted univariable models for each outcome variable (online Supplementary Table 4). We aimed to explore interactions or possible confounding effects to avoid misinterpretations. Then, we conducted a multinomial regression model for each outcome considering class membership as the independent variable to assess the association with several exposures. For each model, the most stable-resilient trajectory was considered the reference category. These multivariable models were additionally adjusted for sex, age, living alone, monthly family income and educational level. Due to potential multicollinearity between some of the exposures we checked the significance and magnitude of correlations through a correlation matrix before running the model (online Supplementary Fig. 3). Regression models were run in 20 imputed datasets and results combined using Rubin's rules (Little and Rubin, 2002).

Additional tests were performed to ensure internal consistency (Cronbach's alpha) and intraclass reliability (intraclass correlation coefficient, ICC) of all the scales in the study, since these were administered in their translated version (Spanish and Catalan). ICC was only calculated for longitudinal assessments (i.e., PHQ-4, UCLA-3 and ‘personal growth’ from SPWB) and limited to pre-pandemic observations.

All statistical analyses were performed in R version 3.6.2 (R Core Team, 2019), and run in RStudio, version 1.3.1093 (RStudio Team, 2020).

## Results

In Table 1 are presented the main characteristics of the total sample (*N* = 5536) at baseline. Our analytical sample was characterised by higher number of females than males (67.39% vs. 32.60%) and by a high proportion of persons with high education (70.82%). The mean age was 51.17 (s.d. = 6.93). From the total sample, 14.43% were living alone, 8.83% were unemployed and 4.11% had a monthly household income lower than 1000€, whereas in 15.93% it was more than 5000€. Moreover, most of the sample (73.80%) was living in an urban area. All scales showed high internal consistencies (Cronbach's alpha ranging from 0.75 to 0.95) and good intraclass reliability (UCLA-3: ICC = 0.75, PHQ-4: ICC = 0.75, ‘personal growth’ from SPWD: ICC = 0.79).

**Table 1.** Main characteristics of the sample at baseline

Characteristics	*N* = 5536	
Sex, *n* (%)		
Male	1805 (32.60)	
Female	3731 (67.39)	
Age, mean (s.d.)	51.17 (6.93)	
Marital status, *n* (%)		
Married	3358 (60.65)	
Single	1015 (18.33)	
Divorced	1029 (18.58)	
Widowed	134 (2.42)	
Living alone (yes), *n* (%)	799 (14.43)	
Educational level, *n* (%)		
Primary education or less	248 (4.49)	
Secondary education	1367 (24.69)	
Higher education	3921 (70.82)	
Occupation, *n* (%)		
Employed	4492 (81.14)	
Unemployed	489 (8.83)	
Retired	555 (10.02)	
Household income, *n* (%)		
<1000€	228 (4.11)	
1000–2000€	1238 (22.36)	
2000–5000€	3188 (57.58)	
>5000€	882 (15.93)	
Living in a city (yes), *n* (%)	4086 (73.80)	
Smoking (yes), *n* (%)	753 (13.60)	Cronbach's *α*
Global health, mean (s.d.)	37.96 (5.62)	0.84
Cognitive function, mean (s.d.)	49.23 (8.97)	0.95
Sleeping problems, mean (s.d.)	8.69 (4.01)	0.68
Personality traits, mean (s.d.)		
Extraversion	31.72 (7.08)	0.85
Emotional stability	33.64 (7.82)	0.88
Agreeableness	41.08 (5.20)	0.78
Conscientiousness	38.31 (6.01)	0.78
Openness to experience	36.23 (6.03)	0.79
Engaged living scale, mean (s.d.)	60.74 (9.35)	0.93
Autonomy, mean (s.d.)	47.35 (1.79)	0.75
Environmental mastery, mean (s.d.)	36.17 (2.28)	0.75
Positive relations with others, mean (s.d.)	27.83 (6.45)	0.84
Brief resilience and coping scale, mean (s.d.)	15.52 (2.38)	0.75
Perceived stress, mean (s.d.)	17.63 (7.25)	0.88

*Note.* The analyses were performed after the multiple imputation, combining 20 imputed datasets using Rubin's rules as described in the ‘Statistical Analysis’ section.

### Mental health trajectories

The first step was to determine the optimal number of latent trajectories according to the fit indices (online Supplementary material Tables from 5 to 7). Although in most outcomes the information criteria (BIC and AIC) pointed to the five- and four-class solutions, the size of the latent classes (<5.00%) and the posterior probabilities (<0.70), lead these solutions to be discarded. Consequently, the 3-class solution provided the best fit. In the case of ‘personal growth’, one of the posterior probabilities was slightly lower than 0.70, but the three-class solution was selected to allow comparability with the other outcomes and to explore this sub-sample characteristics.

**Figure 2.**
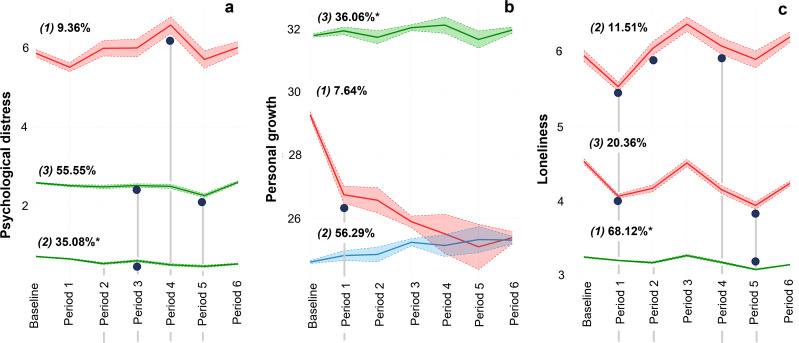
Latent trajectories of different components of mental health. *Note:* The different trajectories were termed as follow: psychological distress (1: ‘chronic’ (*n* = 518), 2: ‘resilient’ (*n* = 1,940), and 3: ‘moderately resilient’ (*n* = 3,072)), personal growth (1: ‘worsening’ (*n* = 423), 2: ‘progressively ascending’ (*n* = 3,116), and 3: ‘resilient’ (*n* = 1,996)), and loneliness (1: ‘resilient – no loneliness’ (*n* = 2,770), 2: ‘chronic – high loneliness’ (*n* = 468), and 3: ‘chronic – medium loneliness’ (*n* = 828)). *Trajectories used as the reference category when multinomial regression models were performed. Blue dots indicate significant changes along the trajectories according to relevant highlights of the pandemic. In particular, we found significant changes in the following periods: period 1 (Spanish Government declared state of emergency), period 2 (beginning of the de-escalation plan), period 3 (Spanish Government declared a new state of emergency), period 4 (notification of a new variant of SARSCoV-2 (VOC B.1.1.7 – Alpha), and started COVID-19 vaccination in Spain), and period 5 (end of the second state of emergency).

In the case of psychological distress (*N* = 5530, see Fig. 2a), we identified a trajectory composed by individuals with PHQ-4 scores above the clinical cut-off pre and during the pandemic. This sub-group was termed ‘chronic’ trajectory (*1*: *n* = 518 (9.36%)) and showed some fluctuations across periods (e.g., there was a significant increase of psychological distress when the de-escalation plan took place (period 2 > period 1: t = 2.383 *p* = 0.017) and with the notification of a new variant of SARS-CoV-2 (VOC B.1.1.7 -Alpha)) (period 4 > period 3: t = 2.869 *p* = 0.004). Conversely, most individuals showed stable trajectories (*2*: n = 1940 (35.08%) and *3*: n = 3072 (55.55%)) across the follow-up period. These trajectories differed essentially in the intercept, but we considered them as ‘resilient’ trajectories according to Bonanno's (2004) definition and were named as ‘resilient’ and ‘moderately resilient’, respectively.

From the three-trajectories of ‘personal growth’ (*N* = 5,535, see Fig. 2b), one group (*3*: *n* = 1996 (36.06%)) was characterised by higher levels of this construct (meaning better perception of personal growth), that was sustained over time, so we termed the ‘resilient’ trajectory. Conversely, we identified another group (‘worsening’ trajectory, *1*: *n* = 423 (7.64%)) that had higher scores before the pandemic and that decreased significantly at the first period of the pandemic (i.e., when the Spanish Government declared the state of emergency; period 1 > pre: *t* = 8.885 *p* < 0.001) and reported a steady and sustained decline over the follow-up. Finally, most of the sample (*2*: *n* = 3116 (56.29%)) was classified into a group (‘progressively ascending’ trajectory) characterised by lower scores at baseline with a slight increase during the studied period. However, this change was not significant and its name was merely descriptive.

Finally, of the three trajectories of loneliness (*N* = 4,066, see Fig. 2c), two of them (*2*: ‘chronic – high loneliness’, *n* = 468 (11.51%), and *3*: ‘chronic – medium loneliness’, *n* = 828 (20.36%)) showed a similar pattern, such that those with higher scores of perceived loneliness before the pandemic showed a decrease at the beginning of the pandemic (i.e., when Spanish Government declared the state of emergency and lockdown was implemented; period 1 > pre: *2 t* = 4.331 *p* < 0.001, *3 t* = 10.329 *p* < 0.001), which increased again in period 2 (when the de-escalation plan began; period 2 > period 1: *2 t* = −4.699 *p* < 0.001, *3 t* = −1.975 *p* = 0.048). From the third period on, there was a decrease until the sixth period, where there was newly an increase in perceived loneliness. Conversely, most of the sample (*1*: ‘resilient – no loneliness’, *n* = 2770 (68.12%)) had low and stable scores during the study-period, meaning low perceived loneliness.

In addition, we calculated the proportions of participants classified in the resilient trajectories of each mental health outcome and the overlap among them. We aimed to see whether those individuals who were resilient in one mental health component were also resilient in the others. Of these results, it should be noted that 65.91% of the participants classified in the trajectory ‘resilient - no loneliness’ were the same individuals as those classified in the trajectories ‘resilient’ and ‘ moderately resilient’ of the psychological distress variable.

### Association between mental health trajectories and exposures

To explore possible interactions or confounding effects among the exposure variables, we performed univariable regression models for each mental health component (online Supplementary Table 4). From these results, highlight the significant associations found in some socio-demographic variables, such as living alone, occupation, household income and educational level, smoking, sleeping problems and some personality traits. These associations largely disappear in the multivariable models when we adjusted for sex, age, living alone, monthly family income and educational level.

In Table 2 the significant results from the multinomial regression models performed for each of the mental health outcomes are presented, expressed as relative risk ratios with 95% confidence intervals (CI). We excluded marital status from the analyses due to a high collinearity (0.72) with the variable living alone (online Supplementary Fig. 3).

**Table 2.** Results from the multivariable models to explore the association between latent trajectory membership and exposures in the mental health constructs

	Psychological distress		Personal growth		Loneliness	
Variables	‘Chronic’	‘Moderately resilient’	‘Worsening’	‘Progressively ascending’	‘Chronic – high loneliness’	‘Chronic – medium loneliness’
Sex						
Male (ref.)	–	–	–	–	–	–
Female	**2.59 (1.91**–**3.51)**	**1.71 (1.48**–**1.99)**	1.07 (0.14–2.45)	**0.82 (0.71–0.95)**	**1.36 (1.02–1.81)**	**1.29 (1.06–1.56)**
Age	**0.95 (0.93–0.97)**	**0.97 (0.96–0.98)**	**1.02 (0.83–1.37)**	**1.02 (1.01–1.04)**	1.00 (0.98–1.02)	0.99 (0.98–1.00)
Living alone						
No (ref.)	–	–	–	–	–	–
Yes	1.32 (0.91–1.91)	0.95 (0.77–1.17)	1.06 (0.77–1.46)	0.91 (0.74–1.11)	**3.15 (2.33–4.28)**	**2.06 (1.63–2.61)**
Occupation						
Employed (ref.)	–	–	–	–	–	–
Unemployed	1.06 (0.68–1.65)	0.96 (0.74–1.25)	1.40 (0.95–2.07)	1.07 (0.83–1.38)	1.09 (0.71–1.67)	1.12 (0.82–1.52)
Retired	0.88 (0.52–1.50)	1.00 (0.77–1.29)	0.95 (0.62–1.45)	0.98 (0.76–1.11)	0.94 (0.59–1.48)	1.27 (0.95–1.72)
Household income						
<1000€ (ref.)	–	–	–	–	–	–
1000–2000€	0.65 (0.35–1.21)	1.15 (0.77–1.71)	1.40 (0.77–2.57)	1.42 (0.99–2.04)	0.91 (0.53–1.57)	1.02 (0.66–1.58)
2000–5000€	0.62 (0.34–1.15)	0.92 (0.62–1.36)	1.35 (0.74–2.46)	**1.52 (1.06–2.17)**	**0.56 (0.31–0.98)**	0.85 (0.54–1.32)
>5000€	0.73 (0.36–1.46)	0.85 (0.56–1.30)	1.03 (0.53–1.99)	1.40 (0.95–2.07)	0.63 (0.33–1.20)	0.75 (0.46–1.22)
Living in a city						
No (ref.)	–	–	–	–	–	–
Yes	1.17 (0.87–1.57)	1.07 (0.92–1.25)	0.88 (0.69–1.12)	1.05 (0.91–1.22)	1.08 (0.82–1.41)	2.06 (0.84–1.22)
Educational level						
Primary education or less (ref.)	–	–	–	–	–	–
Secondary education	0.76 (0.42–1.40)	0.94 (0.65–1.37)	**2.79 (1.11–7.02)**	**0.62 (0.43–0.90)**	0.93 (0.52–1.65)	0.99 (0.61–1.62)
Higher education	0.81 (0.45–1.45)	1.14 (0.79–1.64)	**2.55 (1.02–6.36)**	**0.45 (0.32–0.65)**	1.16 (0.66–2.06)	1.09 (0.69–1.74)
Global health	**0.88 (0.85–0.90)**	**0.94 (0.92–0.96)**	1.00 (0.98–1.03)	**0.97 (0.95–0.98)**	**0.95 (0.92–0.97)**	**0.96 (0.94–0.98)**
Cognitive function	**0.90 (0.89–0.92)**	**0.94 (0.93–0.95)**	1.00 (0.98–1.02)	**0.98 (0.97–0.99)**	**0.97 (0.96–0.99)**	0.99 (0.98–1.00)
Smoking						
No (ref.)	–	–	–	–	–	–
Yes	**1.96 (1.38–2.79)**	**1.27 (1.03–1.56)**	1.07 (0.78–1.47)	1.06 (0.87–1.29)	1.35 (0.99–1.84)	1.02 (0.80–1.30)
Sleeping problems	**1.15 (1.11–1.19)**	**1.07 (1.05–1.10)**	0.97 (0.94–1.00)	0.98 (0.96–1.00)	1.01 (0.98–1.04)	1.01 (0.98–1.03)
Personality traits						
Extraversion	1.00 (0.97–1.02)	0.99 (0.98–1.01)	0.99 (0.97–1.01)	**0.98 (0.97–0.99)**	0.99 (0.97–1.01)	0.99 (0.98–1.01)
Emotional stability	**0.92 (0.89–0.94)**	**0.95 (0.93–0.96)**	0.98 (0.96–1.00)	0.99 (0.98–1.01)	**0.95 (0.93–0.97)**	**0.97 (0.95–0.98)**
Agreeableness	1.02 (0.99–1.06)	**1.01 (1.00–1.03)**	1.00 (0.97–1.03)	0.98 (0.96–1.00)	1.00 (0.97–1.03)	1.00 (0.98–1.02)
Conscientiousness	1.00 (0.97–1.03)	0.99 (0.98–1.01)	0.99 (0.97–1.01)	0.99 (0.98–1.01)	**0.99** (**0.97–1.02)**	0.99 (0.97–1.00)
Openness to experience	**1.03** (**1.00–1.06)**	1.00 (0.99–1.02)	**0.96** (**0.93–0.98)**	**0.95** (**0.94–0.97)**	**1.04** (**1.01–1.06)**	**1.02** (**1.00–1.04)**
Engaged living scale	**0.96** (**0.94–0.98)**	**0.98** (**0.97–0.99)**	0.99 (0.97–1.00)	**0.96** (**0.95–0.97)**	**0.97** (**0.96–0.99)**	**0.98** (**0.96–0.99)**
Autonomy	0.98 (0.92–1.04)	0.98 (0.95–1.02)	1.03 (0.98–1.08)	1.00 (0.96–1.04)	1.03 (0.97–1.11)	0.99 (0.94–1.04)
Environmental mastery	0.98 (0.92–1.05)	1.00 (0.96–1.04)	0.96 (0.90–1.02)	**0.88** (**0.84–0.92)**	1.00 (0.94–1.07)	1.03 (0.98–1.08)
Positive relationships with others	**0.94** (**0.92–0.97)**	0.98 (0.97–1.00)	0.99 (0.97–1.01)	**0.98** (**0.96–0.99)**	**0.86** (**0.84–0.88)**	**0.93** (**0.91–0.94)**
Brief resilience and coping scale	**0.87** (**0.81–0.93)**	0.96 (0.92–1.00)	**0.88** (**0.83–0.95)**	**0.89** (**0.85–0.93)**	**0.91** (**0.86–0.97)**	0.98 (0.94–1.03)
Perceived stress	**1.12** (**1.09–1.15)**	**1.06** (**1.04–1.07)**	1.01 (0.99–1.03)	1.00 (0.99–1.01)	**1.07** (**1.05–1.10)**	**1.04** (**1.02–1.05)**

*Note.* Relative risk ratios (95% CI) from multinomial logistic regression models. Models were run in 20 imputed datasets and results combined using Rubin's rules. Models were adjusted for sex, age, living alone, monthly family income, and educational level. Boldface indicates statistically significant results.

For psychological distress, females, former smokers, having sleeping problems and higher perceived stress, were risk factors to be classified into the ‘chronic’ trajectory but also for the ‘moderately resilient’ trajectory, compared to those in the ‘resilient’ one. Conversely, higher age, better global health and cognitive function, higher emotional stability (personality trait and coping strategies (BRCS), were protective factors for the ‘chronic’ and ‘moderately resilient’ trajectories, taking as a reference the ‘resilient’ class.

In the case of ‘personal growth’, in addition to some similarities, we observed differences in the risk and protective factors of the ‘worsening’ and ‘progressively ascending’ trajectories, compared to the ‘resilient’ class. Regarding similarities, we observed that older age was a risk factor, and that variables such as personality trait ‘openness to experience’ and higher scores on the BRCS (i.e., better resilience and coping strategies) were protective factors. Concerning the differences, those with lower scores in ‘personal growth’ and who experienced a small increase during follow-up (‘progressively ascending’ trajectory), also have as protective factors a better health status, better cognitive function and higher scores in the SPWB scales of ‘positive relations with others’ and ‘environmental mastery’. Conversely, higher and secondary education were risk factors for those classified in the ‘worsening’ trajectory, compared to primary education or less.

As for the loneliness results, we observed similarities between the two trajectories with high scores (‘chronic – high loneliness’ and ‘chronic – medium loneliness’). In both trajectories, variables such as being a female, living alone, and higher perceived stress were risk factors for being classified in these trajectories. Among the protective factors, we found better health status, higher scores on the ‘engagement with life’ and the SPWB ‘positive relations with others’ scales, and in the case of those classified into the ‘chronic – high loneliness’, higher scores on the resilience and coping strategies scale (BRCS), compared to those classified in the ‘resilient – no loneliness’ class.

## Discussion

Mental health during the COVID-19 pandemic attracted much attention, and numerous studies on this topic have been conducted (Salari *et al*., 2020; Prati and Mancini, 2021; Wu *et al*., 2021). However, the vast majority focused on psychological distress as a measure of mental health (Ahrens *et al*., 2021; Batterham *et al*., 2021; Ellwardt and Präg, 2021; Joshi *et al*., 2021; Pellerin *et al*., 2021; Pierce *et al*., 2021; Saunders *et al*., 2021; Shilton *et al*., 2021), which is a conjunction of emotional, psychological and social components (Keyes *et al*., 2020). Our objective was to identify mental health trajectories considering these components as indicators of mental health and to determine whether they were affected in the same way during the different stages of the pandemic. Moreover, we aimed to investigate if the associated variables differed or coincided among the different trajectories.

For the three outcomes studied (psychological distress, personal growth and feelings of loneliness), we identified three latent trajectories. Of these, we differentiated two major trends, a large proportion of people who were in ‘resilient’ trajectories (i.e., better previous functioning with stable trajectories during the follow-up period), and a smaller proportion of participants who were part of ‘chronic-worsening’ trajectories (i.e., low functioning and/or with changes during follow-up). For the ‘resilient’ trajectories, we also observed a lower susceptibility to the changes that occurred in each period of the pandemic, reaffirming Bonanno's (2004) model and the results of research conducted on mental health trajectories during the COVID-19 pandemic (Ahrens *et al*., 2021; Batterham *et al*., 2021; Ellwardt and Präg, 2021; Joshi *et al*., 2021; Pellerin *et al*., 2021; Pierce *et al*., 2021; Saunders *et al*., 2021; Shilton *et al*., 2021). In the case of the so-called ‘chronic-worsening’ trajectories, we observed greater heterogeneity and susceptibility to different periods of the pandemic. For example, regarding psychological distress (emotional component), those participants classified in the ‘chronic’ trajectory had higher scores at baseline than when the state of alarm was declared (period 1), and these scores increased at later points in the pandemic (e.g., period 2, when the de-escalation plan was initiated (‘new normality’); or period 4, when the Alpha variant was reported). However, in the social component (loneliness variable), those people who felt lonelier before the pandemic (chronic - high loneliness), reduced their scores when the state of alarm was decreed (period 1) and home confinement was imposed, returning to their previous scores when the de-escalation and the period of new normality began (period 2).

With respect to psychological distress, one possible explanation for the results obtained is that people classified within this trajectory already had levels of anxious-depressive symptoms above the cut-off point before the pandemic, predisposing them to higher vulnerability. This explanation is further supported by the results of the multinomial regression models, where we observed higher perceived stress as a risk factor and a negative association with higher scores in resilience and coping strategies, and with the personality trait ‘emotional stability’. Our results were in line with previous research. For instance, higher perceived stress during COVID-19 lockdown was found to be a predictor for worse mental health (based on GHQ-28 scores) in a longitudinal study conducted in Germany (Ahrens *et al*., 2021). In the same way, previous mental health diagnosis has been consistently associated to ‘chronic’ or ‘worsening’ trajectories (Pierce *et al*., 2021; Saunders *et al*., 2021), which could be extrapolated to the scores above the PHQ-4 cut-off at baseline in our study. Furthermore, in the investigation conducted by Saunders *et al*. (2021), personality traits such as ‘emotional stability’ was also associated with trajectories with worse anxiety scores (based on the GAD-7), in particular trajectories called ‘moderate/moderately-severe symptoms that become severe over time’ and ‘severe initial anxiety that decreases to normal range, predominantly during lockdown’ (Saunders *et al*., 2021). Taken together, all these factors may be acting synergistically posing these individuals in a more vulnerable situation.

Regarding loneliness, the decrease in scores in the initial period of the pandemic (period 1), was also observed in a previous report by our group, attributing this initial change to the spirit of togetherness that was generated to deal with stay-at-home orders, such as video calls to family and friends or the ‘20:00 h applause’, where thousands of people applauded frontline health professionals from windows or balconies acknowledging them their work and commitment. All these aspects may have helped to intensify social bounds, cooperation and a sense of belonging in the initial stages of the pandemic outbreak. However, in the present study including a much-extended follow-up assessment, indicated that this initial effect declined after the end of home confinement until the initial levels of loneliness were reached (Bartrés-Faz *et al*., 2021). When we characterised these groups of individuals (i.e., ‘chronic – high loneliness’ and ‘chronic – medium loneliness’), we observed that they were mostly females, people who were living alone and individuals with high perceived stress. Unlike for psychological distress, fewer studies have been carried out on loneliness. In much of the research, it has been used as a predictor of mental health and rarely as an outcome (Ahrens *et al*., 2021; Shevlin *et al*., 2023). Studies performed in different countries, that have focused on loneliness during the pandemic, have found somewhat controversial results. Some research found an increase of loneliness during the acute phase of the outbreak (Bu *et al*., 2020; Luchetti *et al*., 2020), whereas other reported a reduction in perceived loneliness in this phase (Bartrés-Faz *et al*., 2021). These findings suggest that the results need to be contextualised, as the effect of the pandemic on loneliness may depend on contextual aspects, such as the restrictions applied in each country.

In the case of the psychological dimension of mental health (‘personal growth’), we identified fewer changes during follow-up, yet some aspects deserve to be mentioned. According to our results, we found that more than half of the sample (those classified in the ‘progressively ascending’ class) had low scores in ‘personal growth’, being people with a feeling of personal stagnation or lack of a sense of improvement or expansion in life. These participants experienced an improvement at follow-up, although not statistically significant. In contrast, a small proportion of the sample (‘worsening’ trajectory) presented a large decrease in scores from the onset of the pandemic (period 1) compared to their baseline scores. Faced with both scenarios, we wondered what variables would be associated with these trajectories to characterise them. In both cases, older age was a risk factor compared to the ‘resilient’ class. This differed from what was found in the literature in studies on emotional distress variables during the pandemic, where younger subjects were more vulnerable (Ellwardt and Präg, 2021; Pierce *et al*., 2021; Saunders *et al*., 2021; Shilton *et al*., 2021). Nevertheless, a review concerning the impact of age on mental health changes during the pandemic found heterogeneous findings in the literature, suggesting that the effect of age may depend on contextual variables but also on the mental health outcome studied (Lebrasseur *et al*., 2021). Our study allows to contextualise these findings in terms of a particular age group (40 to 65 years) and one of the domains of mental health. In addition, both trajectories (‘worsening’ and ‘progressively ascending’), had in common higher resilience and coping strategies, and the personality trait ‘openness to experience’ as positive factors associated to these trajectories. This could be translated into a lower adaptive capacity as well as a tendency towards conservativeness and less openness to experience. However, they differed in a lower risk of being classified in the ‘progressively ascending’ class in the case of better self-reported health, better cognition and higher scores in ‘positive relations with others’ and ‘environmental mastery’, i.e., quality ties to others and the ability to manage complex situations, respectively.

Given these results, and with the calculation of the overlapping of individuals classified in trajectories considered ‘resilient’, we reaffirm our initial hypothesis that the different components of mental health should be analysed separately. We found that within so-called ‘resilience’ there was also heterogeneity, as the proportion of overlapping in the ‘resilient’ individuals among outcomes was only above 50% for psychological distress and loneliness, while for ‘personal growth’ and loneliness it was 26.29%. The greatest overlap, that was found between loneliness and emotional distress, was consistent with that reported in the literature, where both variables have been consistently related (Bu *et al*., 2020; Ahrens *et al*., 2021). Moreover, each outcome was susceptible to different stages of the pandemic and the variables associated with the trajectories presented some differences. These variations included that living alone was only a significant risk factor for loneliness (‘chronic-high/medium loneliness’ trajectories), but not for the other outcomes. Likewise, monthly household income was only related to one of the trajectories of ‘personal growth’ in the adjusted models. Furthermore, lifestyles such as smoking behaviour and sleeping problems were associated with the ‘chronic’ class of the psychological distress measure, which could be related to a maladaptive strategy and a consequence of experienced distress, respectively. For the same class, predictors as ‘emotional stability’ and perceived stress, well-known distress-related variables, were found to be risk factors also for the ‘chronic’ trajectories of loneliness, but not for ‘personal growth’. In addition, from the analysis of the variables associated with the different trajectories, we also observed some similarities. Predictors such as better overall health and better cognitive function were protective factors in all of the studied variables. The relationship between physical and mental health status has been commonly reported in the literature, suggesting a bidirectional relationship (Druss and Walker, 2011). Likewise, anxious-depressive symptomatology has been widely recognised as a risk factor for cognitive impairment (Chodosh *et al*., 2010; Zaninotto *et al*., 2018). Similarly, the personality trait ‘openness to experience’, and some SPWB scales (‘engaged living scale’ and ‘positive relations with others’) were positively associated with better mental health outcomes (i.e., ‘resilient’ trajectories). Finally, emphasise the role of coping strategies, as it was positively associated with those trajectories with better functioning in all the analysed outcomes. Previous research found frequent use of dysfunctional coping strategies and less frequent use of emotion-focused coping strategies in those participants classified into the trajectory ‘high-increasing depressive symptoms’ (Joshi *et al*., 2021). The role of coping strategies is of particular interest as it is a modifiable factor, which can be trained and serve as a preventive strategy for future crises.

From the perspective of practice and policy, our study provides useful information for risk identification. Our research allows to identify and characterise groups of more resilient people and others who are in a situation of chronicity or vulnerability. Furthermore, the fact that we have separated different aspects of mental health (psychological distress, personal growth and feelings of loneliness) and contextualised the fluctuations by considering the relevant events of the pandemic, makes our study of potential great value. In this sense, it allows for the detection of key temporal moments in which to target interventions to strategically prevent to promote a better emotional, psychological and social status. This knowledge could be extrapolated to the current situation, where other social and economic threats have increased, such as the rising price of basic needs (electricity, gas and food), inflation and eventual recession. Exposure to these factors could affect people's health, and the results of these studies could be used to guide preventive strategies.

### Strengths and limitations

The strengths of the present work include a two-year follow-up from the start of the COVID-19 pandemic and the inclusion of baseline information. To the best of our knowledge, no previous study has carried out such a long follow-up (Ahrens *et al*., 2021; Batterham *et al*., 2021; Ellwardt and Präg, 2021; Joshi *et al*., 2021; Pellerin *et al*., 2021; Pierce *et al*., 2021; Saunders *et al*., 2021; Shilton *et al*., 2021). In our study, we analysed data considering the previous two years as the baseline, until February 2022, when the large expansion of the Omicron variant occurred. This is particularly important because, according to Taylor, pandemics are dynamic events and therefore changes in mental health outcomes are expected to occur over time, including a return to baseline levels (Taylor, 2019). This could be observed with a long follow-up and not just at the beginning of the pandemic when lockdown and other covid measures were implemented. Furthermore, we interpreted the fluctuations in the trajectories in terms of the periods of greatest interest for the pandemic, contextualising the changes in the analysed mental health outcomes, suggesting that certain changes might be related to the events taking place in each covid period. This made our study a richer investigation as it was not limited to two major periods (e.g., pre-covid/covid or lockdown/new normality), but allowed us to observe the evolution of psychological, emotional and social outcomes at different points and to identify the most critical moments of the pandemic. Moreover, as we mentioned earlier, we identified trajectories based on proxy measures of different components of mental health, not just psychological distress, since mental health is more than the absence of anxious-depressive symptoms. Therefore, the approach of our study was under Keyes *et al*.'s (2020) definition of mental health and considered emotional, psychological and social elements as indicators of mental health (Keyes *et al*., 2020). The fact that we found differences in trajectories and associated variables among mental health outcomes reinforces our hypothesis and the need for more holistic studies on mental health. Finally, the inclusion of several predictors, such as socio-demographic variables, personality traits, some lifestyles and variables regarding subjective well-being and coping strategies, provided a good overview of the risk and protective factors that characterise each of the trajectories.

However, some limitations deserve to be mentioned. First, we did not use a random sample and it could have introduced some bias limiting the sample representativeness and result generalisability. For example, there was an oversampling of females and participants with higher education. Ideally, we should have fitted the models in a randomised design, but such design is not possible to pursue in the current context. Future research could use post-randomisation techniques based on matching or weighting-based random sampling methods that specifically target potentially varying background characteristics. Secondly, there were differences in the number of observations among periods and variables collected. This fact, although inherent to a longitudinal study, entailed a large number of missingness in most of the predictors, so multiple imputation procedures were performed. In our case the complete case analysis could not be considered due to a drastic reduction of the sample size. Nevertheless, the use of multiple imputation procedures is widely advocated when missing data occur in one or more covariates in a regression model and under an MAR assumption, and in order to ensure the quality of the imputed data, all necessary diagnostics were performed (Sterne *et al*., 2009; White and Carlin, 2010). Thirdly, despite having longitudinal information on some of the exposure variables, multinomial regression models included only baseline scores. Some of these variables, such as occupation, sleep problems, resilience and coping strategies and perceived stress, might have changed during follow-up. Due to differences in the number of observations and the period of collection of each variable, longitudinal analysis was discarded. However, future studies should consider analysing the exposure variables longitudinally, as their possible changes could explain part of the results found. Finally, the identification of trajectories in two of the mental health components was based on screening measures, such as the PHQ-4 and the UCLA-3. While much of the research in this field has used these or similar measures (Bu *et al*., 2020; Fancourt *et al*., 2021; Pierce *et al*., 2021; Saunders *et al*., 2021; Shevlin *et al*., 2023), researchers and policymakers should be aware of the accuracy limitations with such tools, and interpret the results with caution.

## Data Availability

The authors encourage interested investigators to reach out and we will honour all reasonable and scientifically motivated requests for data access and make the raw data available when required.

## Supplementary material

For supplementary material accompanying this paper visit http://doi.org/10.1017/S2045796023000136. click here to view supplementary material
