# Supplementary material for: COVID-19 after two years: trajectories of different components of mental health in the Spanish population
Source: Epidemiol Psychiatr Sci. 2023 Apr 17;32:e19. doi: 10.1017/S2045796023000136 (PMC10130737; doi:10.1017/S2045796023000136)
Supplement: Supplementary file 1 [file S2045796023000136sup001.docx]

**Supplementary material**

**Supplementary table 1.** STROBE statement checklist for observational studies

|  | | Item No | Recommendation | Page |
| --- | --- | --- | --- | --- |
| **Title and abstract** | | 1 | (*a*) Indicate the study’s design with a commonly used term in the title or the abstract | Title sheet |
|  |  |  | (*b*) Provide in the abstract an informative and balanced summary of what was done and what was found | Title sheet |
| Introduction | | | |  |
| Background/rationale | | 2 | Explain the scientific background and rationale for the investigation being reported | 1-4 |
| Objectives | | 3 | State specific objectives, including any prespecified hypotheses | 4 |
| Methods | | | |  |
| Study design | | 4 | Present key elements of study design early in the paper | 4,5 |
| Setting | | 5 | Describe the setting, locations, and relevant dates, including periods of recruitment, exposure, follow-up, and data collection | 5, figure 1 |
| Participants | | 6 | (*a*) *Cohort study*—Give the eligibility criteria, and the sources and methods of selection of participants. Describe methods of follow-up | 4,5 |
|  |  |  | (*b*) *Cohort study*—For matched studies, give matching criteria and number of exposed and unexposed | N/A |
| Variables | | 7 | Clearly define all outcomes, exposures, predictors, potential confounders, and effect modifiers. Give diagnostic criteria, if applicable | 6-9 |
| Data sources/ measurement | | 8* | For each variable of interest, give sources of data and details of methods of assessment (measurement). | *6-9* |
| Bias | | 9 | Describe any efforts to address potential sources of bias | 9-11 |
| Study size | | 10 | Explain how the study size was arrived at | 5, 9 |
| Quantitative variables | | 11 | Explain how quantitative variables were handled in the analyses. If applicable, describe which groupings were chosen and why | 6-9 |
| Statistical methods | | 12 | (*a*) Describe all statistical methods, including those used to control for confounding | 9-11 |
|  |  |  | (*b*) Describe any methods used to examine subgroups and interactions | 10-11 |
|  |  |  | (*c*) Explain how missing data were addressed | 10, supplementary table 3, supplementary figures 1 and 2 |
|  |  |  | (*d*) *Cohort study*—If applicable, explain how loss to follow-up was addressed | 9 |
|  |  |  | (*e*) Describe any sensitivity analyses | N/A |
| Results | | | | Page |
| Participants | 13* | (a) Report numbers of individuals at each stage of study—eg numbers potentially eligible, examined for eligibility, confirmed eligible, included in the study, completing follow-up, and analysed | | N/A |
|  |  | (b) Give reasons for non-participation at each stage | | N/A |
|  |  | (c) Consider use of a flow diagram | | N/A |
| Descriptive data | 14* | (a) Give characteristics of study participants (eg demographic, clinical, social) and information on exposures and potential confounders | | 11 and table 1 |
|  |  | (b) Indicate number of participants with missing data for each variable of interest | | Supplementary table 3 |
|  |  | (c) *Cohort study*—Summarise follow-up time (eg, average and total amount) | | Figures 1 and 2 |
| Outcome data | 15* | *Cohort study*—Report numbers of outcome events or summary measures over time | | 11-13, figure 2 |
| Main results | 16 | (*a*) Give unadjusted estimates and, if applicable, confounder-adjusted estimates and their precision (eg, 95% confidence interval). Make clear which confounders were adjusted for and why they were included | | Supplementary table 4, supplementary figure 3, page 11 |
|  |  | (*b*) Report category boundaries when continuous variables were categorized | | Table 2, supplementary table 4 |
|  |  | (*c*) If relevant, consider translating estimates of relative risk into absolute risk for a meaningful time period | | N/A |
| Other analyses | 17 | Report other analyses done—eg analyses of subgroups and interactions, and sensitivity analyses | | Supplementary materials |
| Discussion | | | |  |
| Key results | 18 | Summarise key results with reference to study objectives | | 15 |
| Limitations | 19 | Discuss limitations of the study, taking into account sources of potential bias or imprecision. Discuss both direction and magnitude of any potential bias | | 21, 22 |
| Interpretation | 20 | Give a cautious overall interpretation of results considering objectives, limitations, multiplicity of analyses, results from similar studies, and other relevant evidence | | 15-22 |
| Generalisability | 21 | Discuss the generalisability (external validity) of the study results | | 21, 22 |
| Other information | | | |  |
| Funding | 22 | Give the source of funding and the role of the funders for the present study and, if applicable, for the original study on which the present article is based | | 23 |

**Supplementary table 2.** Results from the mixed effects models

| **Psychological distress (PHQ-4)** | | | | | | | |
| --- | --- | --- | --- | --- | --- | --- | --- |
|  | AIC | BIC | logLik | deviance | Chisq | Df | Pr(>Chisq) |
| Random intercept only | 125663 | 125696 | -62827 | 125655 |  |  |  |
| Random slope only | 138801 | 138835 | -69397 | 138793 | 0 | 0 |  |
| Random slope only | 138801 | 138835 | -69397 | 138793 |  |  |  |
| Random intercept and slope | 125069 | 125119 | -69528 | 125057 | 13736 | 2 | <0.0001 |
| Random intercept only | 125663 | 125696 | -62827 | 125655 |  |  |  |
| Random intercept and slope | 125069 | 125119 | -62528 | 125057 | 598.21 | 2 | <0.0001 |
| **Personal growth** | | | | | | | |
|  | AIC | BIC | logLik | deviance | Chisq | Df | Pr(>Chisq) |
| Random intercept only | 88501 | 88531 | -44246 | 88493 |  |  |  |
| Random slope only | 95322 | 95353 | -47657 | 95314 | 0 | 0 |  |
| Random slope only | 95322 | 95353 | -47657 | 95314 |  |  |  |
| Random intercept and slope | 88403 | 88449 | -44195 | 88391 | 6923.1 | 2 | <0.0001 |
| Random intercept only | 88501 | 88531 | -44246 | 88493 |  |  |  |
| Random intercept and slope | 88403 | 88449 | -44195 | 88391 | 101.75 | 2 | <0.0001 |
| **Loneliness (UCLA)** | | | | | | | |
|  | AIC | BIC | logLik | deviance | Chisq | Df | Pr(>Chisq) |
| Random intercept only | 74672 | 74705 | -377332 | 74664 |  |  |  |
| Random slope only | 81076 | 81109 | -40534 | 81068 | 0 | 0 |  |
| Random slope only | 81076 | 81109 | -40534 | 81068 |  |  |  |
| Random intercept and slope | 74443 | 74492 | -37215 | 74431 | 6637.2 | 2 | <0.0001 |
| Random intercept only | 74672 | 74705 | -37332 | 74664 |  |  |  |
| Random intercept and slope | 74443 | 74492 | -37215 | 74431 | 233.23 | 2 | <0.0001 |

**Supplementary table 3.** Percentage of missingness in the exposures

| **Variables** | **% of missingness** |
| --- | --- |
| Sex | 0.00% |
| Age | 0.00% |
| Marital status | 0.02% |
| Living alone | 0.02% |
| Educational level | 0.02% |
| Occupation | 0.02% |
| Household income | 0.00% |
| Living in a city | 0.02% |
| Global health | 0.11% |
| Cognitive function | 0.11% |
| Sleeping problems | 0.07% |
| Smoking | 0.07% |
| Personality traits | 38.62% |
| Engaged living scale | 12.19% |
| Autonomy | 13.82% |
| Environmental mastery | 13.82% |
| Positive relationships | 13.82% |
| Brief resilience and coping scale | 39.17% |
| Perceived stress | 48.15% |

**Supplementary figure 1.** Density plots for continuous variables conducted to check the imputation quality


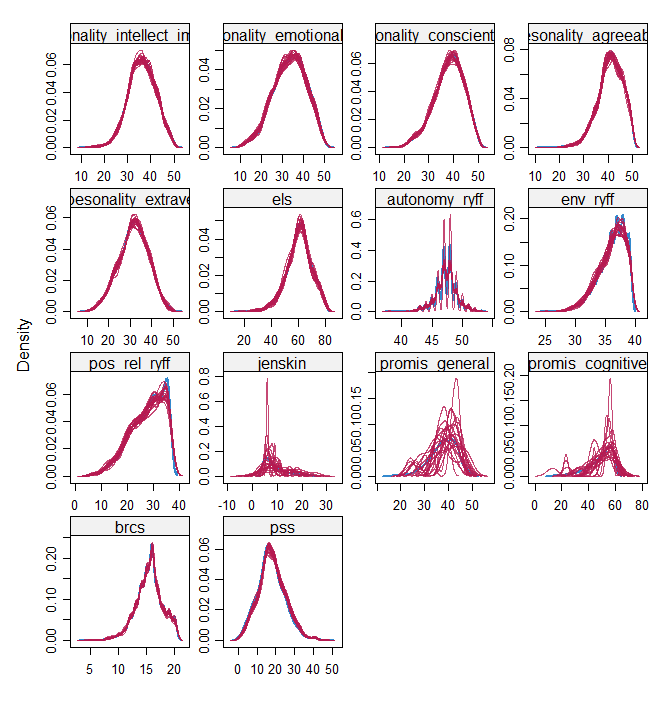


**Supplementary figure 2.** Stripplots for categorical variables conducted to check the imputation quality


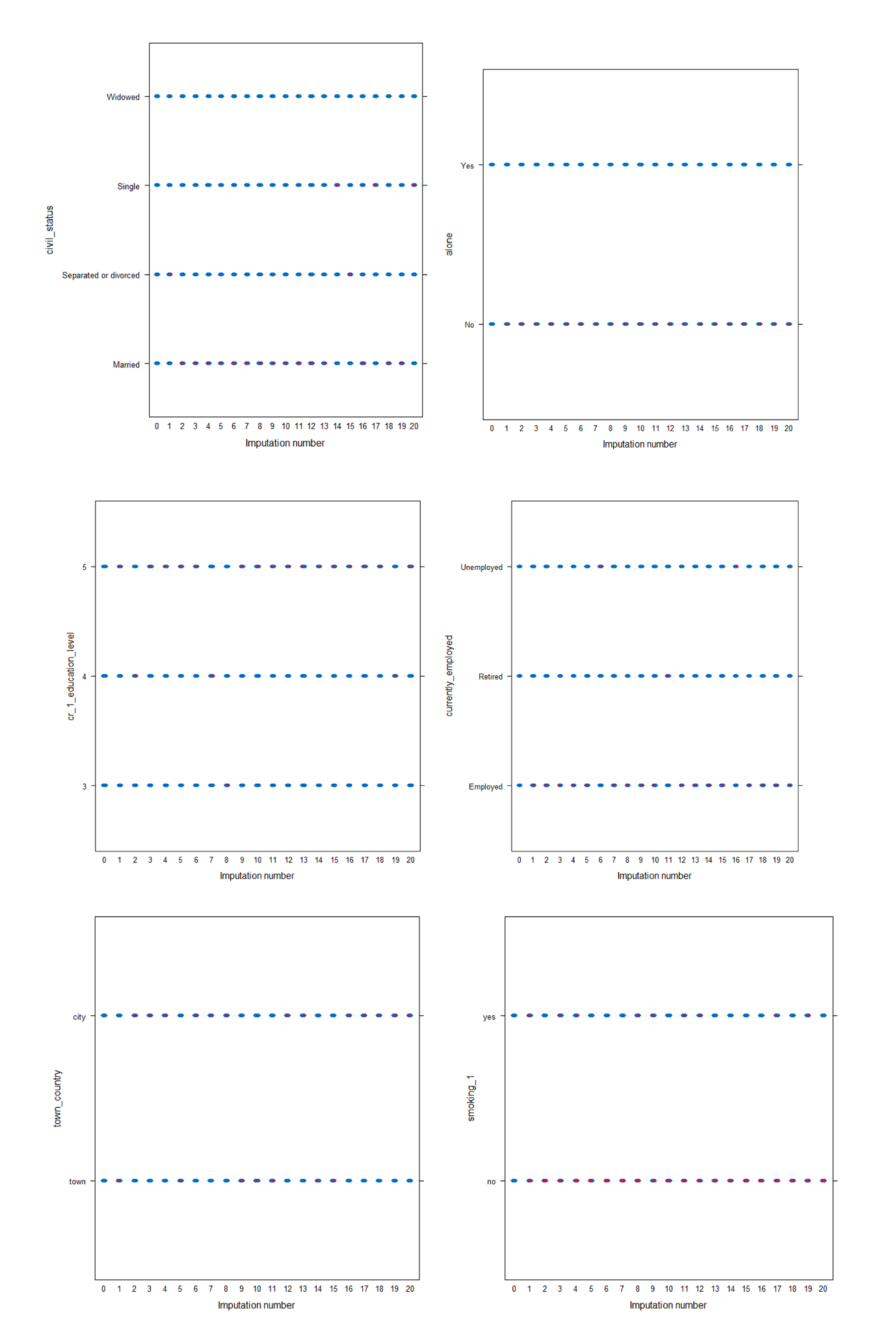


**Supplementary figure 3.** Correlation matrix to assess multicollinearity among the predictors


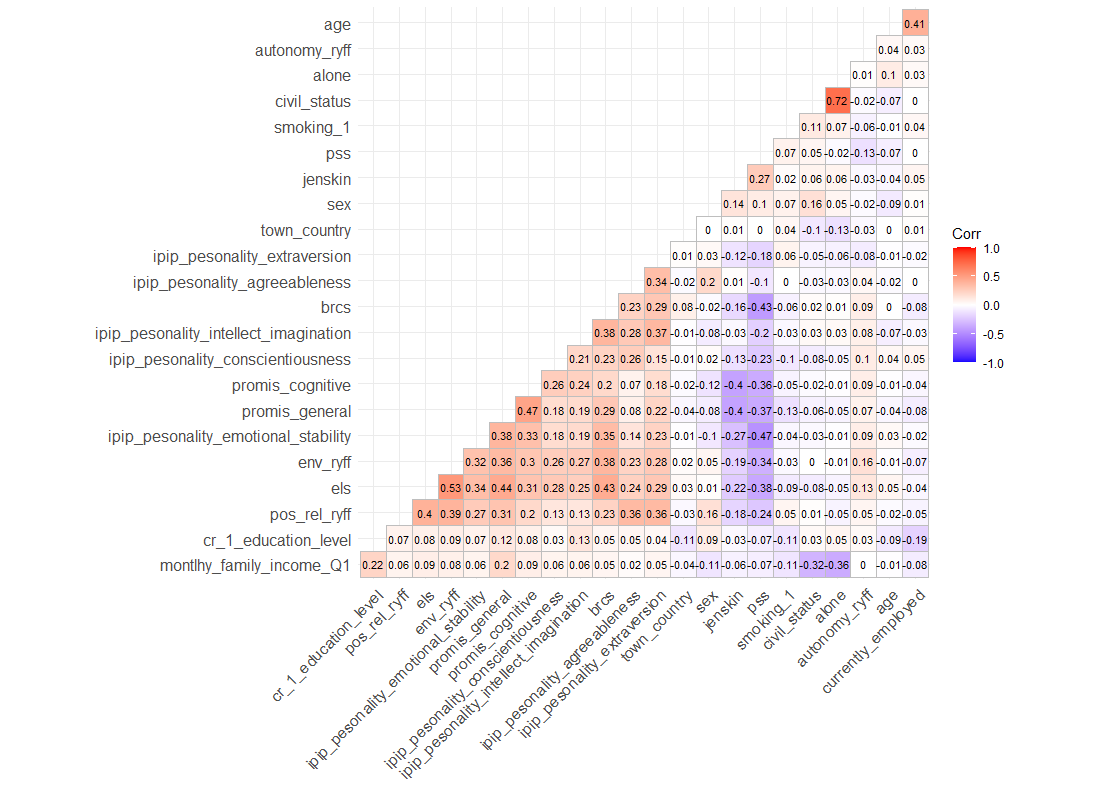


**Supplementary table 4.** Results from the univariable models to explore the association between latent trajectory membership and exposures in the mental health constructs

|  | **Psychological distress** | | **Personal growth** | | **Loneliness** | |
| --- | --- | --- | --- | --- | --- | --- |
| **Variables** | **“Chronic”** | **“Moderately resilient”** | **“Worsening”** | **“Improving”** | **“Chronic – high loneliness”** | **“Chronic – medium loneliness”** |
| Sex |  |  |  |  |  |  |
| Male (ref.) | - | - | - | - | - | - |
| Female | **2.66 (2.11-3.34)** | **1.86 (1.65-2.09)** | 1.13 (0.89-1.43) | **0.81 (0.71-0.91)** | **1.26 (1.01-1.57)** | **1.26 (1.07-1.49)** |
| Age | **0.95 (0.94-0.97)** | **0.97 (0.96-0.98)** | **1.02 (1.00-1.03)** | **1.02 (1.01-1.03)** | 0.99 (0.98-1.00) | 0.99 (0.98-1.00) |
| Living alone |  |  |  |  |  |  |
| No (ref.) | - | - | - | - | - | - |
| Yes | **1.45 (1.12-1.87)** | 1.02 (0.86-1.20) | 1.08 (0.80-1.44) | 0.93 (0.79-1.09) | **2.98 (2.40-3.71)** | **1.99 (1.63-2.42)** |
| Occupation |  |  |  |  |  |  |
| Employed (ref.) | - | - | - | - | - | - |
| Unemployed | **1.73 (1.28-2.35)** | 1.07 (0.87-1.32) | 1.40 (0.98-2.00) | **1.25 (1.02-1.54)** | **1.57 (1.16-2.12)** | 1.29 (0.99-1.68) |
| Retired | **0.61 (0.42-0.87)** | **0.73 (0.61-0.88)** | 1.29 (0.90-1.86) | **1.48 (1.21-1.80)** | 0.91 (0.64-1.29) | 1.12 (0.88-1.43) |
| Household income |  |  |  |  |  |  |
| <1000€ (ref.) | - | - | - | - | - | - |
| 1000-2000€ | **0.57 (0.38-0.88)** | 1.26 (0.91-1.75) | 1.30 (0.73-2.32) | 1.24 (0.92-1.67) | 0.73 (0.49-1.08) | 0.91 (0.62-1.35) |
| 2000-5000€ | **0.32 (0.21-0.48)** | 0.93 (0.68-1.27) | 1.18 (0.68-2.06) | 1.10 (0.82-1.46) | **0.31 (0.21-0.45)** | **0.61 (0.41-0.89)** |
| >5000€ | **0.21 (0.13-0.33)** | **0.65 (0.46-0.91)** | 0.84 (0.46-1.54) | 0.78 (0.57-1.07) | **0.25 (0.16-0.39)** | **0.43 (0.28-0.65)** |
| Living in a city |  |  |  |  |  |  |
| No (ref.) | - | - | - | - | - | - |
| Yes | 1.05 (0.84-1.31) | 1.03 (0.90-1.17) | 0.92 (0.73-1.17) | 1.05 (0.92-1.19) | 1.08 (0.87-1.34) | 1.02 (0.86-1.21) |
| Educational level |  |  |  |  |  |  |
| Primary education or less | - | - | - | - | - | - |
| Secondary education | **0.53 (0.35-0.82)** | 0.89 (0.65-1.21) | **2.74 (1.07-7.05)** | **0.61 (0.43-0.84)** | 0.67 (0.43-1.03) | 0.88 (0.57-1.37) |
| Higher education | **0.41 (0.28-0.62)** | 0.88 (0.65-1.17) | **2.10 (0.83-5.32)** | **0.35 (0.26-0.49)** | **0.60 (0.40-0.91)** | 0.82 (0.54-1.23) |
| Global health | **0.73 (0.71-0.74)** | **0.86 (0.85-0.87)** | **0.96 (0.94-0.98)** | **0.90 (0.89-0.91)** | **0.84 (0.82-0.85)** | **0.90 (0.89-0.91)** |
| Cognitive function | **0.83 (0.82-0.84)** | **0.90 (0.89-0.91)** | 0.98 (0.97-1.00) | **0.94 (0.94-0.95)** | **0.92 (0.91-0.93)** | **0.95 (0.95-0.96)** |
| Smoking |  |  |  |  |  |  |
| No (ref.) | - | - | - | - | - | - |
| Yes | **2.24 (1.74-2.89)** | **1.37 (1.15-1.63)** | 1.12 (0.82-1.52) | **1.18 (1.00-1.40)** | **1.49 (1.17-1.90)** | 1.12 (0.91-1.39) |
| Sleeping problems | **1.39 (1.35-1.42)** | **1.18 (1.15-1.20)** | 0.99 (0.97-1.02) | **1.05 (1.03-1.06)** | **1.15 (1.13-1.18)** | **1.08 (1.06-1.10)** |
| Personality traits |  |  |  |  |  |  |
| Extraversion | **0.92 (0.90-0.93)** | **0.96 (0.95-0.97)** | **0.97 (0.95-0.98)** | **0.93 (0.92-0.94)** | **0.92 (0.90-0.93)** | **0.96 (0.95-0.97)** |
| Emotional stability | **0.81 (0.79-0.82)** | **0.90 (0.89-0.91)** | **0.96 (0.95-0.98)** | **0.94 (0.93-0.95)** | **0.88 (0.86-0.89)** | **0.93 (0.92-0.94)** |
| Agreeableness | **0.96 (0.94-0.99)** | 0.99 (0.97-1.00) | **0.97 (0.95-0.99)** | **0.92 (0.91-0.94)** | **0.93 (0.91-0.95)** | **0.97 (0.96-0.98)** |
| Conscientiousness | **0.90 (0.89-0.92)** | **0.95 (0.94-0.96)** | **0.97 (0.95-0.99)** | **0.94 (0.93-0.95)** | **0.93 (0.92-0.95)** | **0.96 (0.95-0.97)** |
| Openness to experience | **0.93 (0.91-0.95)** | **0.96 (0.95-0.97)** | **0.93 (0.91-0.95)** | **0.90 (0.89-0.91)** | **0.96 (0.95-0.98)** | 0.99 (0.97-1.00) |
| Engaged living scale | **0.87 (0.85-0.88)** | **0.93 (0.92-0.94)** | **0.96 (0.95-0.98)** | **0.91 (0.91-0.92)** | **0.90 (0.89-0.91)** | **0.94 (0.93-0.95)** |
| Autonomy | **0.85 (0.80-0.90)** | **0.92 (0.89-0.95)** | 1.00 (0.94-1.06) | **0.93 (0.90-0.96)** | 0.95 (0.90-1.00) | **0.94 (0.91-0.98)** |
| Environmental mastery | **0.64 (0.61-0.67)** | **0.81 (0.78-0.83)** | **0.86 (0.82-0.91)** | **0.71 (0.69-0.74)** | **0.73 (0.70-0.76)** | **0.85 (0.82-0.88)** |
| Positive relationships with others | **0.87 (0.85-0.88)** | **0.94 (0.93-0.95)** | **0.97 (0.95-0.99)** | **0.92 (0.91-0.93)** | **0.83 (0.82-0.84)** | **0.91 (0.90-0.92)** |
| Brief resilience and coping scale | **0.64 (0.61-0.67)** | **0.82 (0.79-0.84)** | **0.82 (0.77-0.86)** | **0.74 (0.72-0.77)** | **0.74 (0.70-0.77)** | **0.87 (0.84-0.90)** |
| Perceived stress | **1.27 (1.24-1.30)** | **1.12 (1.11-1.13)** | **1.04 (1.02-1.06)** | **1.06 (1.05-1.07)** | **1.15 (1.13-1.17)** | **1.07 (1.06-1.09)** |

*Note.* Relative risk ratios (95% CI) from multinomial logistic regression models. Models were run in 20 imputed datasets and results combined using Rubin’s rules. Boldface indicates statistically significant results.

**Supplementary table 5.** Model fit indices for different number of classes solutions for psychological distress (PHQ-4)

| No. of classes | Loglik | npm | AIC | BIC | Entropy | class 1 (%) | class 2 (%) | class 3 (%) | class 4 (%) | class 5 (%) | Average posterior probabilities |
| --- | --- | --- | --- | --- | --- | --- | --- | --- | --- | --- | --- |
| 1 | -62528.364 | 6 | 125068.728 | 125108.435 | 1 | 100 | NA | NA | NA | NA | 1.000 |
| 2 | -61695.263 | 10 | 123410.526 | 123476.705 | 0.60 | 11.482 | 88.517 | NA | NA | NA | 0.872  0.903 |
| **3** | **-61374.635** | **14** | **122777.270** | **122869.92** | **0.54** | **9.367** | **35.081** | **55.551** | **NA** | **NA** | **0.860**  **0.742**  **0.805** |
| 4 | -61295.112 | 18 | 122626.224 | 122745.347 | 0.48 | 27.142 | 6.636 | 34.122 | 32.097 | NA | 0.590  0.866  0.737  0.687 |
| 5 | -61278.940 | 22 | 122601.881 | 122747.476 | 0.47 | 4.249 | 31.320 | 34.394 | 23.743 | 6.292 | 0.812  0.585  0.734  0.565  0.605 |

**Supplementary table 6.** Model fit indices for different number of classes solutions for personal growth

| No. of classes | Loglik | npm | AIC | BIC | Entropy | class 1 (%) | class 2 (%) | class 3 (%) | class 4 (%) | class 5 (%) | Average posterior probabilities |
| --- | --- | --- | --- | --- | --- | --- | --- | --- | --- | --- | --- |
| 1 | -44195.484 | 6 | 88402.969 | 88442.682 | 1 | 100 | NA | NA | NA | NA | 1.000 |
| 2 | -44029.113 | 10 | 88078.227 | 88144.415 | 0.55 | 70.551 | 29.448 | NA | NA | NA | 0.911  0.714 |
| **3** | **-44009.436** | **14** | **88046.872** | **88139.536** | **0.48** | **7.642** | **56.296** | **36.061** | **NA** | **NA** | **0.618**  **0.812**  **0.719** |
| 4 | -43987.555 | 18 | 88011.110 | 88130.249 | 0.56 | 1.607 | 59.548 | 36.224 | 2.619 | NA | 0.665  0.788  0.746  0.655 |
| 5 | -43984.202 | 22 | 88012.404 | 88158.019 | 0.61 | 55.447 | 0.090 | 3.541 | 1.282 | 39.638 | 0.793  0.688  0.644  0.659  0.744 |

**Supplementary table 7.** Model fit indices for different number of classes solutions for loneliness (UCLA)

| No. of classes | Loglik | npm | AIC | BIC | Entropy | class 1 (%) | class 2 (%) | class 3 (%) | class 4 (%) | class 5 (%) | Average posterior probabilities |
| --- | --- | --- | --- | --- | --- | --- | --- | --- | --- | --- | --- |
| 1 | -37215.358 | 6 | 74442.716 | 74480.578 | 1 | 100 | NA | NA | NA | NA | 1.000 |
| 2 | -35390.855 | 10 | 70801.711 | 70864.815 | 0.68 | 70.388 | 29.611 | NA | NA | NA | 0.915  0.933 |
| **3** | **-35239.470** | **14** | **70506.940** | **70595.286** | **0.68** | **68.125** | **11.510** | **20.363** | **NA** | **NA** | **0.899**  **0.849**  **0.768** |
| 4 | -35239.470 | 18 | 70514.940 | 70628.527 | 0.43 | 65.592 | 0.00 | 11.510 | 22.897 | NA | 0.519  NA  0.849  0.728 |
| 5 | -35239.470 | 22 | 70522.940 | 70661.769 | 0.35 | 0.000 | 0.000 | 11.510 | 61.288 | 27.201 | NA  NA  0.849  0.346  0.661 |
